# A brief review of some artificial intelligence methods in nephrology

**DOI:** 10.1007/s00467-025-06995-9

**Published:** 2025-10-27

**Authors:** Kevin V. Lemley

**Affiliations:** Department of Pediatrics, Keck School of Medicine, University of Southern California, Los Angeles, CA USA

**Keywords:** Machine learning, Nephropathology, Convolutional neural net, Large language model, Chatbot, Computational pathology

## Abstract

This brief, focused review considers two of the more commonly used artificial intelligence (AI) methods encountered in nephrology publications: machine vision based on convolutional neural networks (CNNs) and chatbots, such as ChatGPT, based on large language models. It is intended to offer a mostly non-technical, intuitive understanding of these methods, including their uses and limitations. CNNs have been used for some time to segment and classify important features of digitized kidney biopsy images. In addition to the identification of pathologic primitives, CNN approaches may be used to predict so-called sub-visual features of biopsies, such as kidney survival rates. Large language models are newer players in the medical AI field. Although seemingly easy to use as natural language tools, most currently available chatbots have been characterized by inconsistent performance, hallucinations, and even a higher CO_2_-footprint than CNN-based models.

**Graphical abstract:**

**Figure:**
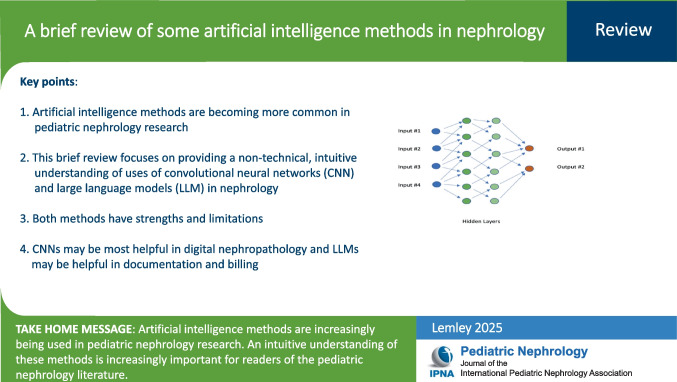
A higher resolution version of the Graphical abstract is available as Supplementary information

**Supplementary Information:**

The online version contains supplementary material available at 10.1007/s00467-025-06995-9.

## Introduction

I assume that most readers are not experts in machine learning, so I offer this as a brief and focused primer about digital pathology using machine learning and the performance of large language models (LLMs) and chatbots in medicine. The goal is not to allow the readers to apply these techniques by themselves, but to better and more critically understand their capabilities and limitations when reading the nephrology literature. This is offered in the spirit in which we would wish most pediatric nephrologists to know the basics about next-generation sequencing or CRISPR technology [1]. A more general treatment of artificial intelligence in glomerular disease has previously appeared in this journal [2].

Machine learning (ML) is a subfield within the general subject of artificial intelligence, a form of computational science useful for analyzing extremely large data sets. “Learning” and “intelligence” may inevitably evoke the idea of consciousness or personality, but I see ML as a tool and not as a person. Thus, I judge it according to its utility and not its “motives” or “intent,” if one can even speak about these in this context. This very practical approach means I am not bothered by one of the common complaints about ML, the black box criticism. A black box is a system for which we know the input and the output, but nothing about how the former leads to the latter. This is not an entirely apt description of ML in any event. First, we do know how the ML algorithms work. Someone originally designed them after all! They are simply too complex for humans to intuitively understand, much like the insides of your computer, which most of us can effectively use, nevertheless. This is like people talking about high-dimensional spaces. Although our 3-dimensional intuitions do not help us to *visualize* 10-dimensional space, we can use straightforward mathematics to work quite well with such a space, prove things about it, etc. In the case of ML, the ultimate test of its utility is whether it works for its given task. This means we must rigorously validate the accuracy and utility of the algorithm’s performance in any context. The capacity of ML models has increased dramatically over the last two decades, both because of the increasingly large digital databases used for training and the use of faster algorithms and processing devices such as graphics processing units (GPUs), originally developed for digital image processing in video gaming.

## Feature learning

A classic example of machine learning is representation or feature learning [3]. This is used on classification or feature detection tasks by training systems to recognize specific features in raw data, typically based on supervised learning. Examples might be recognizing and labeling glomeruli in the digital image of a kidney biopsy, or something as prosaic as a machine recognizing hand-written zip codes on an envelope at the post office. *Supervised learning* occurs using pre-labeled inputs, classified in advance by a gold-standard expert and reflecting the *ground truth*. In addition to supervised learning, there are also *unsupervised* forms of ML, typically used on unlabeled data for purposes of pattern recognition, simplification (e.g., dimensionality reduction) and clustering of data, and semi-supervised learning, in which learning is based on a mix of labeled and unlabeled training data.

## A primer on artificial neural networks

**Fig. 1:**
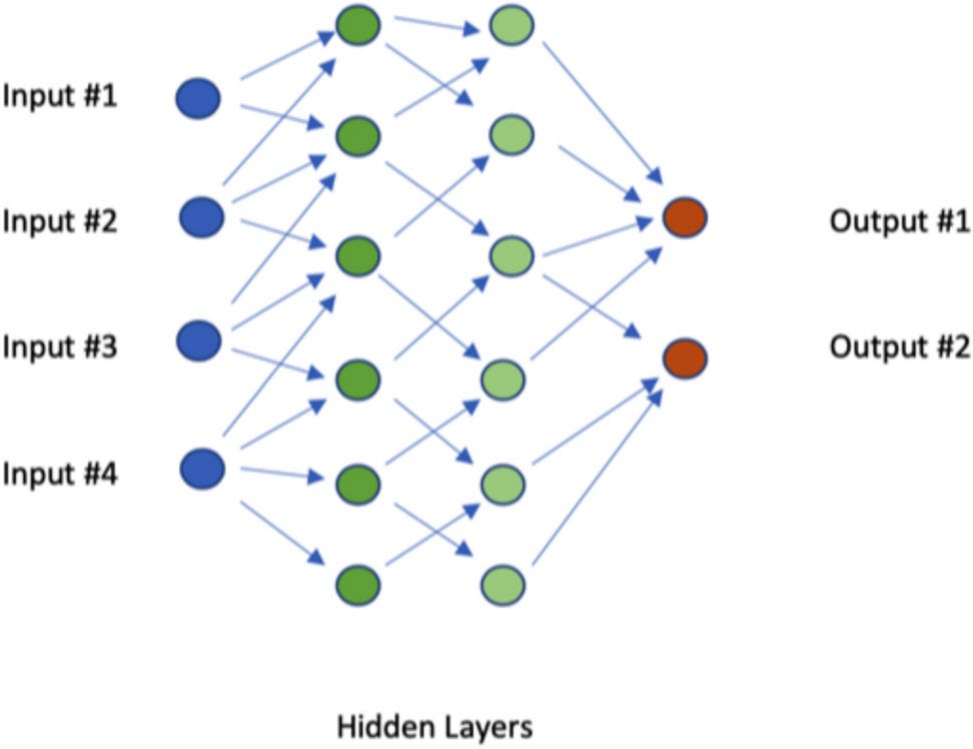
Schematic representation of a simple, multi-layer neural net. The input layer (blue dots) may represent any type of data, for example, part of a digital image of a kidney biopsy. This layer feeds into a network of connected hidden layers of nodes (green dots) with each node receiving (weighted) input from one or more nodes in a preceding layer. After the intermediate hidden layers, the final output layer (red dots) represents the distribution of different classes, for example, whether the input represents a normal glomerulus or an abnormal glomerulus

**Fig. 2:**
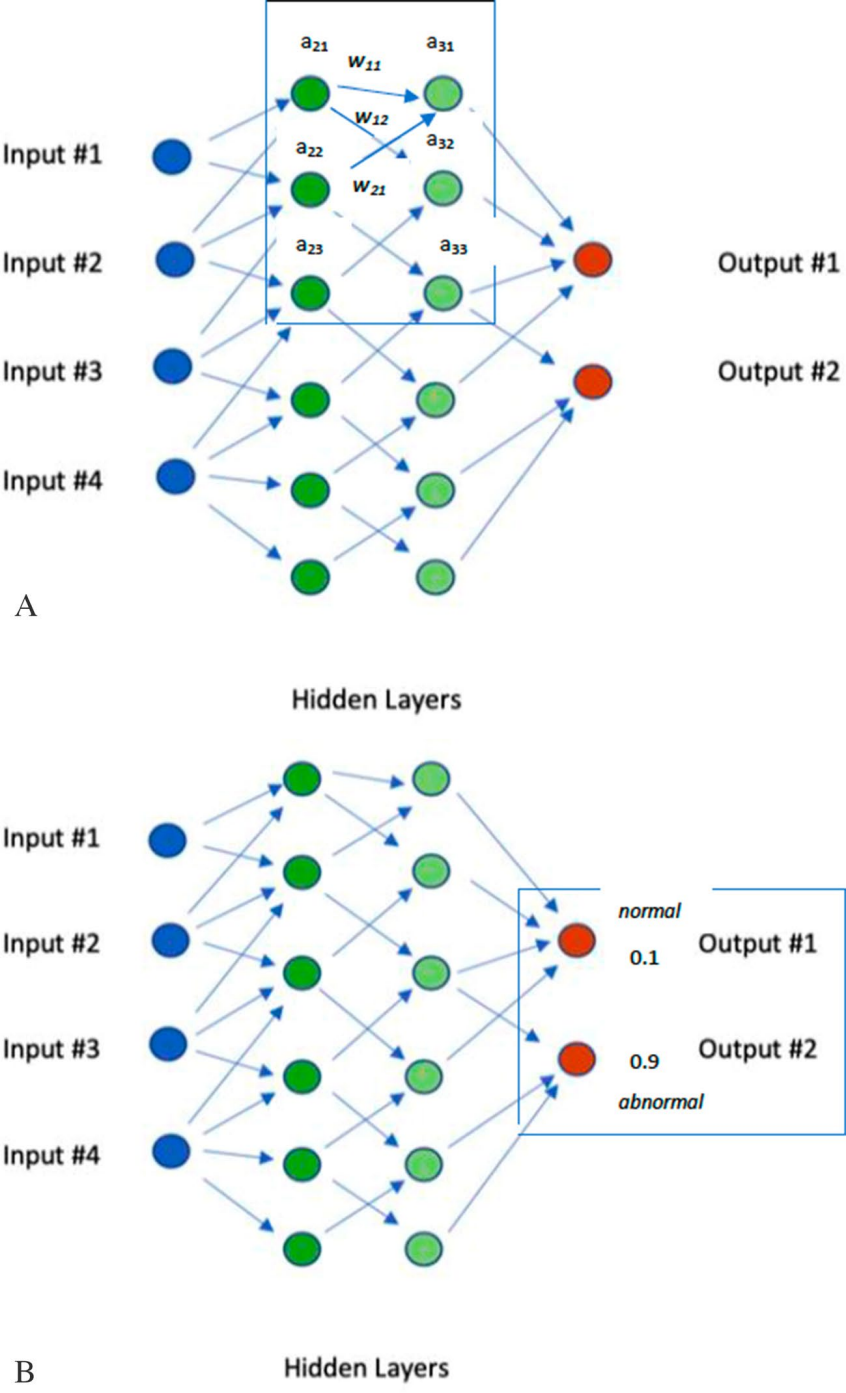
**A** Close-up of Fig. 1 illustrating (in box) relations of node values (*a*_11_, etc.) and their weightings (*w*_11_, *w*_12_, etc.). Most weightings and node values left out for clarity. Relations illustrated by example: *a*_32_ = *w*_12_·*a*_21_ + *w*_31_·*a*_23_. **B** Close-up of Fig. 1 illustrating (in box) relations of output values to the ground truth and calculation of mean-squared error cost function. If the ground truth for the outcomes is abnormal (here designated by a value of 1), the mean-squared error of the actual output is [(0.9)^2^ + (0.1)^2^]/2 = [0.81 + 0.01]/2 = 0.41. Notice that the error cost is disproportionately influenced by value of Output #1, despite it having a small weight

A common architecture used for supervised ML is an artificial neural network (Fig. 1). These are analogous to biological neural networks and involve a processing system connecting signals from input nodes (or neurons) to output nodes via a system of intermediate *hidden* nodes. If there are multiple hidden layers of nodes in the model, these are often referred to as *deep learning models*. In Fig. 1, the output nodes (red dots) may represent feature classes to which the input data are assigned. Unlike the input/output nodes, the activity states of hidden nodes are not (directly) observable. As with biological neurons, an intermediate node may receive numerous inputs from previous nodes as well as having numerous outputs to subsequent nodes in the net. The activities of the nodes in the preceding layer are weighted regarding their influence on subsequent node activation (Fig. 2A). In addition, a fixed bias signal may be added to the sum of the weighted inputs to a node (subsequently referred to simply as “weightings”). Since images are invariant to spatial translation, that is, they look the same if they are shifted right or left, parameters such as weightings can be shared across blocks of image pixels, lowering the number of unique parameters required to be specified for the model. The sensitivity of a node to activation is generally non-linear with respect to its weighted inputs. Non-linearity allows the net to recognize complex patterns in data. A common *activation function* is the rectified linear unit (ReLU), which zeros out negative inputs but passes on and does not saturate for positive inputs (unlike logistic, binary step, sigmoid, or hyperbolic activation functions, for example). Non-linear filters such as ReLU help to make filters adaptable and limit vanishing gradients during backward propagation (see below).

The passage of signals through the net from values of training data in the input layer, for example, through the hidden layers to the output layer is referred to as *forward propagation*. Once the output values are compared against the pre-labeled correct output values, a *cost function* (also called an error or loss function) can be calculated which tells us how different the outputs were from the gold-standard values in terms of a maximum likelihood model or a mean squared error. Different forms of cost functions may be appropriate to different types of data and applications. Mean squared error, for example, may be an appropriate cost function for linear regression models with normally distributed errors. In Fig. 1, assume the inputs are image components (pixels) of a glomerulus in the digital image of a kidney biopsy section and the outputs are a classification of the glomerulus as either normal or abnormal. Assume after the first run forward through the network (with the initial node weightings), the output node for normal has a value of 0.1 and the output node for abnormal has a value of 0.9. These may be thought of as representing the likelihood that the glomerulus should be classified either as normal or abnormal. These must add up to 1.0 (100%), since the glomerulus must be either abnormal or normal. Say that the ground truth is that the glomerulus is abnormal. From this information, the cost function representing the error in classification may be calculated (Fig. 2B). This is where the learning comes in to machine learning. Much like human learners, such systems may be said to *learn with supervision* in the form of comparing predicted values of the output feature vector with the pre-established gold-standard values of that vector.

## Convolutional neural networks

The type of ML most often used for the analysis of visual data (e.g., digital images of kidney biopsies) involves a *convolutional neural network*, or CNN. A commonly encountered machine learning architecture for non-visual data is the *random forest*, which is based on an ensemble of decision trees and is used, for example, in diagnosis classification or event (such as AKI or intradialytic hypotension) prediction [4]. “Convolution” refers to a specific type of connectivity between nodes within some of the hidden layers of the neural net. In contrast to fully connected layers, in which all the nodes of the prior layer connect with all the nodes in the next layer, in CNNs, an input or preceding layer undergoes convolution (consider it as a fancy form of multiterm multiplication) with a filter layer (kernel) to mix neighboring pixel values of a subset of the preceding layer. This occurs over a subset (tiles) of all the nodes in the preceding layer as the kernel slides across the input data, performing element-wise multiplication of the input values with the kernel elements followed by adding the products. This yields a single output value for each input value of the input layer. Typical tiling domains are 3 × 3, 5 × 5, or 7 × 7 pixels. By sharing weighting factors across tiles, the total number of weightings in a layer is limited, helping to reduce overfitting risks. The computed output of a convolution layer (the filtered version of the preceding input layer) is called a *feature map* and represents specific features like edges and textures in the preceding layer that are used for object detection or segmentation by the CNN. Convolution layers tend to increase the number of subsequent nodes; another type of connectivity occurs in pooling layers, which combine or average values of neighboring pixels to reduce the dimensional output of a layer.

CNNs are often described as being analogous to the (mammalian) visual system from the retina to the visual cortex. From direct excitation of retinal cells (equivalent to pixels on a digital image), multiple levels of integrative neural connections (starting within the retina itself) eventually lead to the activation of a neuron in the visual cortex that corresponds to a specific higher-level image. This neuron is often called the “grandmother” cell and is activated only by inputs that correspond to the retinal image of your grandmother. Hence, a large number of specific retinal activation events, processed through the network, lead to a simple classification outcome: grandmother or not-grandmother, depending on whether the final node is activated or not.

*Training* the network (i.e., learning) consists of trying to minimize classification error (Fig. 2B) based on making changes in the node output weightings in the hidden layers. These changes are determined by calculating the slope or gradient of the cost function going backward through the network (*back propagation*) after a round of forward propagation. Just like with calculus, the minimum of a function can be found by identifying its stationary points, the *x* points for which the function’s slope is zero. In this case, the *x* is a vector, whose dimension depends on the number of weighting parameters that affect the error function. Once the “topographic landscape” of the cost function gradient has been established by back propagation, various methods of efficiently locating minima of the various parameters (e.g., stochastic gradient descent [5]) can be used to tweak the weighting parameters in the direction opposite to the gradient—the steepest descent—and thus decrease the value of the cost function. By comparison with labeled (annotated) training data, the model can determine how good its “guess” was and learn from it, even if it initially randomly assigned weightings to the nodes of the hidden layers. Each run forward through the network allows backpropagation to refine the changes in node weightings. The learning performance of the neural net can be influenced by choices of a number of variable *hyperparameters*, which allow for model fine-tuning. These can be optimized by trials over a subset of all the data (hold-out data) that are not subsequently used in net training to avoid overfitting. One example of a hyperparameter is the step size (learning rate) of gradient descent, how big our steps are down the slopes of the cost function landscape during our search for minima in the error. Increasing the learning rate improves the rate of convergence of training to an optimum weighting configuration, however possibly at the cost of “jumping over” and missing global minima in the cost function during gradient descent. Specific gradient descent algorithms help avoid settling in a local, rather than a global, minimum.

## Model validation

A well-trained model should do a good job of predicting outcome classifications or scores, for example, when applied to a new dataset, different from the one on which it was trained. This is referred to as generalizability. One way to enhance generalizability is by rigorous model *validation*. The simplest and cleanest method of model validation is to divide the totality of all training data at the beginning of model development into three random samples: a small hold-out set for hyperparameter refinement, a larger set for model derivation/development, and a final validation test set. An alternative approach is any of various forms of cross-validation, in which the overall data set is repeatedly randomly re-partitioned into training and validation sets. One common strategy is leave-one-out cross-validation, in which, from a data set of *n* values, *n*−1 values are used for training, and then the model is tested on how well it classifies the remaining held-out data point. A total of *n* such tests are done. Model accuracy can be evaluated from the mean-squared error of all the tests. A computationally less demanding alternative is k-fold cross-validation.

### Saliency mapping

**Fig. 3:**
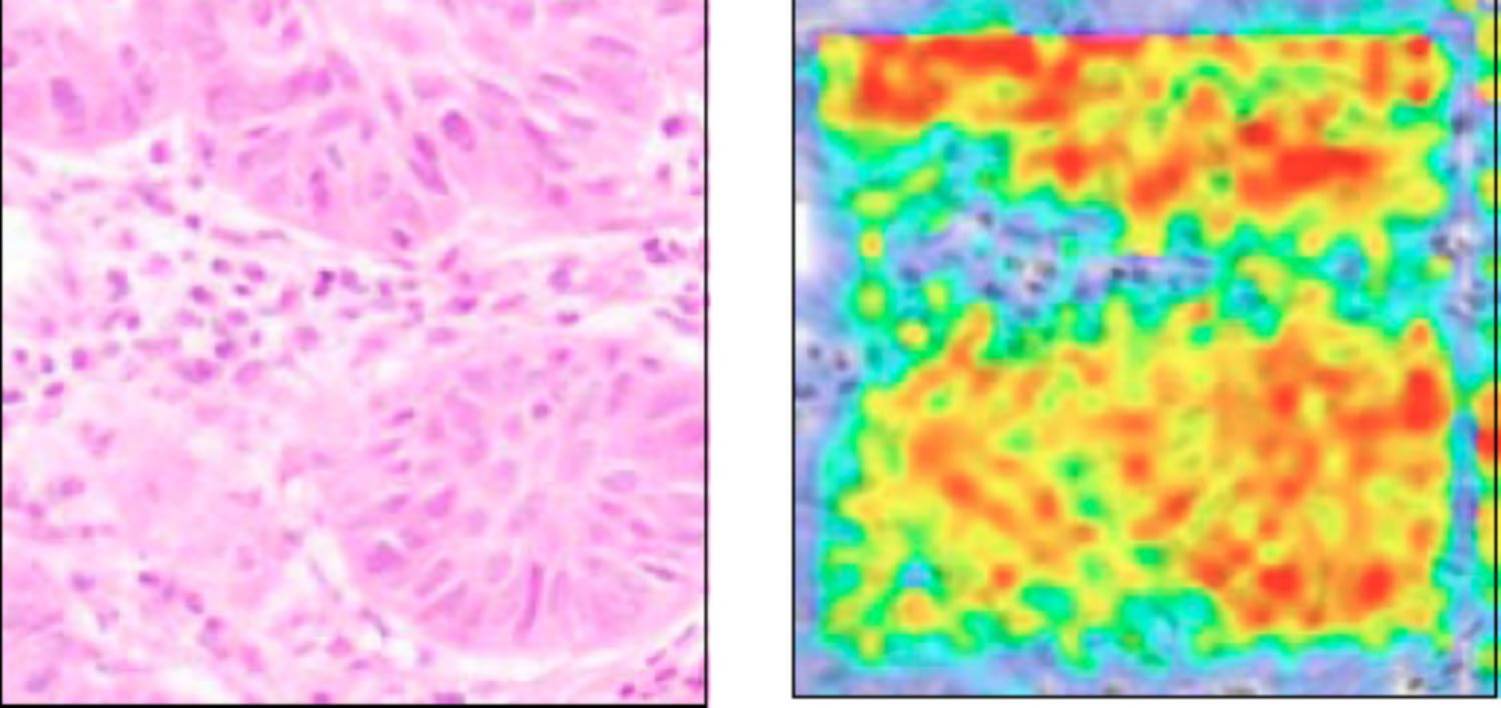
Example of an image of a hematoxylin and eosin-stained skin cancer biopsy (left) and the same tissue (right) imaged as a gradient-weighted class activation mapping (Grad-CAM), a type of pixel-based heat map saliency mapping. The hotter colors represent parts of the original image that are more important for the classification task of the CNN. From Fig. 6 in Sauter D, Lodde G, Nensa F et al. (2022) Validating automatic concept-based explanations for AI-based digital histopathology. Sensors (Basel) 22:5346. https://doi.org/10.3390/s22145346. The source article was published Open Access and use of the figure is allowed

Saliency maps are ML tools that highlight those parts of the input image that most strongly influence the CNN’s classification output. This in some ways allows us a look inside the proverbial black box. Saliency maps can be constructed in a number of different ways, but basically, they follow a backpropagation-type approach to identify, through changes in the derivative of the class score, those pixels with the greatest influence on the scores. They are related to neural models of visual attention and are typically represented as a heat map (Fig. 3). As a form of source attribution, they may, for example, help instruct us in previously unrecognized parts of a biopsy image that are important for diagnosis or prognosis, and perhaps even yield mechanistic insights.

## Challenges to neural net methods

### Overfitting

**Fig. 4:**
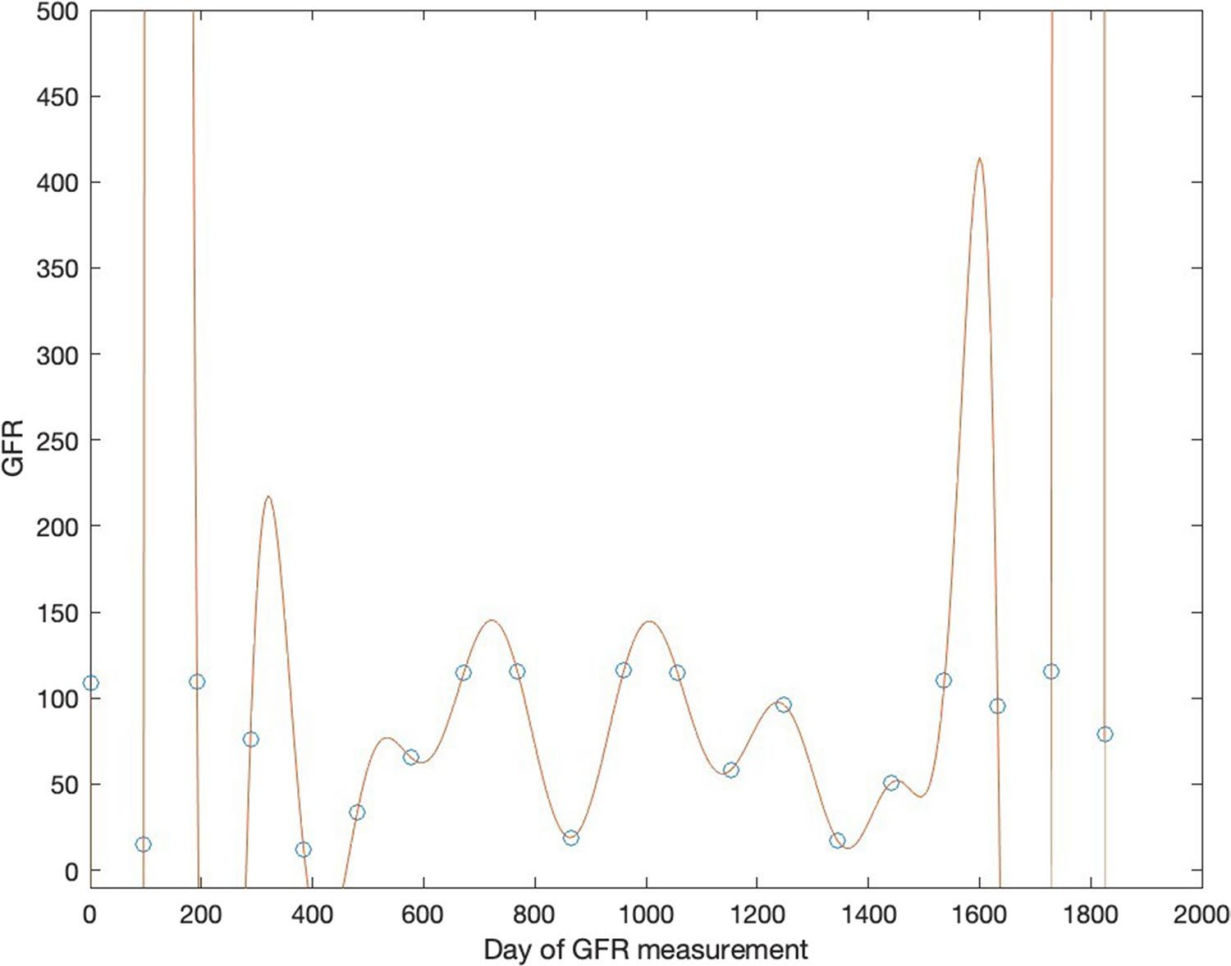
Polynomial of degree 19 describing GFR as a function of time (GFR = *a*_0_ + *a*_1_*T* + … + *a*_18_*T*^18^ + *a*_19_*T*^19^, where *T* is time in days of the simulated GFR measurement, marked with an open circle). The fit curve was calculated using the Matlab® function polyfit (T,GFR,19)

A typical deep learning architecture may have many hidden layers and many hundreds or thousands of initial input neurons. If the connectivity between layers is high (each preceding neuron sending signals to several subsequent neurons), the number of potential neuron-neuron weightings may be in the tens or hundreds of thousands. Even after sharing local weightings among input nodes, this gives an immense computational capacity to the neural net. It is something like fitting a polynomial equation to a data variable when you can use arbitrarily high powers of that variable in the equation. This leads to a problem much bigger than that of a black box, namely *overfitting*. Consider Fig. 4. In this graph, a polynomial of degree 19 in time (days) is fit to a set of 20 randomly generated values of GFR. As long as the polynomial equation is of degree 19 or higher, the fit of the polynomial curve to the data can be perfect. At the same time, although the curve perfectly fits all the 20 quite feasible GFR values, it is “off the charts” in 3 different ranges (e.g., 100–200 days) and gives negative GFR values in 2 different time ranges (e.g., 200–300 days).

This is an aspect of what is referred to as the *curse of dimensionality*: the number of distinct configurations of a neural net increases exponentially with the number of variables or parameters. Models generally can be made to fit data better by adding more adjustable parameters. Although this flexibility improves the ability to fit a model to training data, it raises the risk of overfitting and thus lowers the generalizability of the fitted model to new data. One can help avoid overfitting by applying a *regularization* procedure to the cost function, for example, by adding a penalty term which increases with the complexity of the model. The elastic net loss function, for example, combines penalties from both ridge regression and lasso regression.

## Bias in ML

As with any statistical estimation methods, socioeconomic, gender, or racial biases existing in the training data are easily baked into the resultant models. Testing of models on diverse patient populations can help to mitigate this risk [1]. Added countervailing bias in weightings may be used to compensate for a known bias in the training population.

## Uses of CNN in digital nephropathology

The earliest and still one of the most common uses for CNNs in digital pathology is *segmentation*, which automatically “paints” regions of digital images according to their tissue identity (typically by a color overlay). It is commonly used to automatically identify and locate specific objects, such as glomeruli. This can be valuable when the total number of glomeruli [6] or their volumes is considered informative, as in some predictive models. If large amounts of tissue are to be evaluated (as in necropsy), there may be theoretical time advantages to bypassing human assessment. Another advantage of ML biopsy analysis is the possibility of greater consistency of annotation in comparison to human evaluators, which may be especially valuable in the case of large multi-center studies. CNNs for this use are often presented as valid when they agree with expert annotators over 90% of the time in flagging glomeruli. This seems like a low bar, given that recognition of hand-written zip codes by scanners at the post office is not considered useful if success rates are not over 98%. A novel, recently described potential use of ML segmentation in kidney biopsy analysis [7] is real-time web-based evaluation of biopsy adequacy from smartphone digital images, reducing dependence on evaluation of cores by a pathologist on site.

Another useful feature in digital nephropathology is estimating the degree of interstitial fibrosis and/or tubular atrophy (IFTA), which is a particularly powerful predictor of eventual progression to kidney failure. IFTA is typically graded semi-quantitatively by kidney pathologists: < 25%, 25–50%, 50–75%, and > 75%. Although quantitative estimates of IFTA can be obtained by point-counting methods [8], stereological and morphometric methods can be quite labor-intensive when compared to ML methods. In addition to identifying pathologic “primitives” (such as glomeruli or IFTA), CNNs can also detect so-called *sub-visual* features—abstract features, such as the 5-year kidney survival rate, which are not even visible to a human observer. Training a CNN to predict GFR slope from annotated data is, in fact, no more difficult than training one to identify/segment glomeruli, classify them as normal or abnormal, or estimate IFTA from digital kidney biopsy images. Kolachalama and colleagues [9] used six CNN models on digital images of kidney biopsies from 171 patients to predict a number of clinical features of the patients, including 1-, 3-, and 5-year kidney survival; they compared the classification performance of the CNNs with models based on fibrosis scores extracted from corresponding clinical biopsy reports. For the kidney survival rates, the CNNs had areas under the curve (AUC) between 0.87 and 0.90, whereas the pathologist-estimated fibrosis scores had AUCs between 0.78 and 0.81.

### Sparse data

Other impressive examples of predictive performance by CNNs have been reported in other disease states. For example, Esteva and colleagues [10] trained a CNN on 129,450 annotated images of human skin lesions and showed AUCs from 0.94 to 0.96 in distinguishing cases of squamous cell carcinoma from seborrheic keratoses. Similarly, Ting and colleagues [11] trained a CNN on 76,370 annotated retinal images and achieved an AUC of 0.96 in detecting diabetic retinopathy. How were Kolachalama and colleagues able to achieve the performance they did on a much smaller training set? They used an approach called *transfer learning*. They started with a CNN that had already been trained to recognize 1000 different object classes (planes, trains, cats, dogs, etc.) from over a million annotated images (Google Inception v3 architecture in their case) and then *retrained* the latter layers of the neural net on their kidney biopsy images. Thus, they did not “waste” their valuable biopsy image data training the first, non-specific layers of the CNN (which might just be detecting general contrasts and image edges). There is no inherent reason that transfer learning needs to be limited to sequential application of just two training regimens. For example, one might re-train the Google Inception v3 architecture to predict various morphometric features in biopsy images that are known to be predictive of eGFR changes over time [8] and then subsequently re-retrain the CNN to predict the slope of GFR from the same digital biopsy images. It is possible that the model accuracy would be enhanced by multiple transfers of learning. Unsupervised ML identifying predictive visual features of biopsy using a bag-of-words approach has also shown good performance (an AUC of 0.91) in predicting loss of eGFR at 1 year [12].

Other examples of applications of CNNs include disease classification. Yang and colleagues [13] emulated the usual workflow of a nephropathologist by combining several ML techniques built on a pre-trained CNN architecture, starting with two-stage models for glomerulus detection from whole slide images (WSI) of 1379 single light microscopy kidney biopsy slides from three different medical centers, followed by a 5-disease category diagnostic classification and finally classification of 6 different glomerular lesions in cases of class II and III lupus nephritis. Validation based on 60 hold-out slides was performed at each stage of the workflow. The numbers of CNN-identified glomeruli exceeded the numbers from the biopsy reports, suggesting sensitive detection. Performance was assessed in terms of the balanced F score (the harmonic mean of the recall, the positive prediction sensitivity, and precision, the positive prediction accuracy). The F score for different medical centers and slide stains generally ranged from 0.9 to 0.94 for glomerulus detection, 0.7 to 0.94 for disease classification (with significant variation of performance among centers and disease classes), with AUCs for lupus nephritis glomerular lesion classification from 0.7 to 0.94. The authors also applied class activation mapping to examples of the specific glomerular lesions to highlight which parts of the glomerular image were important for identifying the lesions. The authors suggest that such a ML-based tool could streamline and support the workflow of the nephropathologist.

Like the use of sub-visual features of kidney biopsy images to make a kidney disease diagnosis, *retinal* images from diabetic patients may underlie making a kidney disease diagnosis. Dong and colleagues [14] developed a multi-modal transformer-based supervised deep learning model to distinguish pure diabetic nephropathy (DN) from non-diabetic renal disease (NDRD, including NDRD + DN) based on retinal images (as well as pixel-level retinal lesion-segmented maps) and clinical data from 246 adult patients with type 2 diabetes and biopsy-proven CKD (DN or NDRD). There was a 20% hold-out internal validation set from the 246 subjects, as well as an additional internal prospective cohort and multi-institutional external cohort validation sets. As with Yang et al. [13], visualization maps provided attribution to those retinal lesions that contribute the most to a diagnosis of DN (in this case, the pixel-wise lesion segmentation). Model performance was assessed via AUCs, which were generally quite high (> 0.95); *F*_1_ was also high (> 0.9), other than for the external validation groups. Performance was also somewhat less in a cohort of subjects with fundus photographs taken at non-standard angles. The ML model was clearly superior in distinguishing DN from NDRD compared to the assessment of a panel of three nephrologists for a cohort of 44 patients from the internal validation set.

## Chatbots and large language models (LLMs)

Another form of ML that has recently gained widespread interest in medicine is chatbots based on large language models [15–17]. LLMs have become more popular since the release of OpenAI’s Generative Pretrained Transformer, ChatGPT, as a generative search engine. A transformer is a deep learning machine architecture in which, for example, a string of natural language text (referred to as a “token”) is converted to another text string under a certain probability distribution based on a massive *corpus* of unlabeled text, providing a powerful tool in *natural language processing*. This form of generative AI was initially used for language translation but can be applied to images, sounds, videos, etc. As a generative search engine, an LLM typically converts a query token to an answer token, possibly along with associated sources for the answer. In this, LLMs differ significantly from the ML models used with CNNs. The latter are trained based on quantitative agreement of predicted outcomes with previously annotated and objectively measurable examples (the ground truth), while the former are developed based on the syntactic plausibility of the neighboring text based on the training corpus token distribution. Thus, LLMs in this pure form have no dependency on even the existence of a ground truth.

Despite the company’s name, the architecture of ChatGPT is so far not generally open-source. The same is true for Google’s chatbot, Gemini (formerly Bard), Anthropic’s Claude, or the chatbot Perplexity. That means their net architecture and training data are not publicly available. This presents a significant challenge for vigorous (as opposed to anecdotal) validation of model performance by independent investigators, a core expectation of experimental sciences. The new Chinese release (DeepSeek®) was released with weighting parameters and training data. However, most assessments of DeepSeek’s performance in the medical literature are still based on post facto comparisons to presumed ground truth (e.g., answering medical examination questions) and against the performance of competing LLMs. Even with respect to summarizing scientific publications—one of the presumed strengths of LLMs—most of the more common LLMs tested have shown a fairly strong susceptibility to generalization bias [18]. Training on a corpus of “scraped,” publicly available online data presents another challenge. This data may be publicly available, but it is not necessarily open access in the sense of copyright law, and the practice of using it has been under legal challenge.

One of the more colorful limitations to chatbots based on LLMs is the high frequency of *hallucinations* in their answers to queries [15]. For example, when used as search engines to answer user queries, only about half of apparently informative responses are fully supported by citations [19]. In addition, only about 75% of supplied supporting citations actually support the answer proposed. Non-existent but convincing publications in support of statements may be offered in seemingly perfect PubMed format. Possibly due to competing learning strategies used for attaining fluency and accuracy, it has been reported that the accuracy of citations supporting an answer to a chatbot query may be *inversely related* to the fluency and perceived utility (as judged by human evaluators) of the model outputs [19]. Another concerning finding with ChatGPT is a report of inconsistent changes in model performance (in opposite directions!) of GPT-3.5 and GPT-4 over a relatively short time span (3 months) of presumed continued model development. Chen and colleagues [20] found that over a short set of four tasks characterized by relative objectivity and ease of evaluation—for example, answering the question of whether a 5-digit number is prime or not—the accuracy of GPT-4 decreased from 97.6% to 2.4% over 3 months, while the accuracy of GPT-3.5 increased from 7.4% to 86.8%. The accuracy of computer code generation (not over 50% in any case) dropped over time for both GPTs. It has been suggested that this instability of performance in the face of model development may be an inherent and unavoidable flaw for LLMs [16].

In one study [21], the performance of any of the tested LLMs on clinical cases from pediatric nephrology was not good. For example, hallucinations were common, dangerously wrong treatments were occasionally recommended, and none of the LLMs had an accuracy that would likely allow clinical application. Medical LLMs surprisingly performed even worse than generalist models, such as ChatGPT. Although minor improvements in performance were achieved in one model using *retrieval-augmented generation*, based on adding adjunctive domain-specific training data to an LLM, this may just be due to biasing the model recommendations more toward the consensus knowledge base (guidelines and publications) of the human sources of expert ground truth. More restricted application of chatbots to simpler tasks based on a more limited target output, such as ICD coding and billing, may show better performance [22].

### Reasoning systems

There seems to be an increasing rate of hallucinations with the most recent generation of chatbots. DeepSeek-R1 and OpenAI o3 are examples of *reasoning AI models* designed to avoid this. Reasoning systems add an inference engine, which can perform logical operations, and ontological and semantic networks to the base corpus of scraped knowledge. These chatbots may try to improve accuracy by adding extra steps, such as trying multiple methods or double-checking an answer. Literally, such steps may be as strange as asking the chatbot if the answer it just gave to a query is actually true or having multiple LLMs check the answers of each other. Any of these “solutions” to the problem of chatbot hallucinations seems at least a little problematic.

This raises the question of just what constitutes an acceptable hallucination rate. This clearly depends on the consequences of acting on an erroneous but otherwise convincing answer to a query. Can saliency mapping be used to put the brakes on hallucinations? Perhaps if a heat map of saliency is not broad or strong enough, it could flag a chatbot answer as problematic. The ultimate safeguard against hallucinations is the responsibility of the knowledgeable human end-user to verify the appropriateness and accuracy of the chatbot output. For overworked, time-stressed clinical practices today, this may be challenging to assure.

## Conclusion

I have tried in this primer to transmit an intuitive understanding of two of the more popular applications of artificial intelligence to medicine. As is probably clear from my presentation, I hold much greater hope for the utility of convolutional neural nets, e.g., in computer vision applications, than I do for large language models. This derives from the fact that the former are anchored in a ground truth that can be established objectively in advance, alternatively, of semantics besting syntax. The greatest weakness of CNN is overfitting (the curse of dimensionality), for which several mitigating strategies are available. For simple, real-time medical applications (e.g., interpreting radiographs), CNNs may be more accurate than LLMs. In addition, and increasingly recognized to be important for AI, their superior computational efficiency means that their CO_2_-footprint is also probably superior to LLMs [23]. Chatbots based on LLMs are evaluated fundamentally by their fluency rather than on any informational content. Generally, this can only be judged post facto and subjectively. A single correct answer to a question (with the possible exception of yes/no questions) cannot in general be established in advance, due to the plasticity of English and other natural languages. Despite their current limitations, the massive investment of resources by several technology companies makes it hard to totally rule out future useful advances using LLMs in medicine.

## Supplementary Information

Below is the link to the electronic supplementary material.

Graphical abstract (PPTX 140 KB)

## Glossary

*Activation function*: Function describing the relation between the sum of the input signals to a node and its output signal. Examples include the identity, ReLU, and sigmoid functions.
*Back propagation*: Algorithm by which information describing the cost function flows backward through the computational network in order to determine the gradient of the cost function with respect to its adjustable parameters to train the network.
*Convolutional neural network*: Type of neural network often used for analysis of visual or spatial data with multiple layers of nodes connected via convolutional (a type of multiterm value multiplication through a filter, or kernel), pooling or fully connected architectures.
*Cost function*: Also called an error or loss function. Quantifies how far the network outputs deviate from the gold-standard, ground truth values. Changes in adjustable parameters of the network to minimize the cost function optimize model performance.
*Curse of dimensionality*: The challenge presented to modeling in a space of relatively high-dimensionality (high degrees of freedom) based on a relatively small set of training and test data
*Feature map*: Output of a convolutional layer through kernel multiplication which represents various features (e.g. edges or contrasts) of the input image.
*Forward propagation*: Progress of input values into a network, through the hidden layers, to the final output values for a single cycle.
*Ground truth*: Accurate, gold-standard data (generally independently verified) used to train and test a machine learning model.
*Hidden nodes*: Nodes in a deep neural net that are not directly observable, that is, are neither input nodes nor output nodes of the net.
*Hyperparameter*: Tunable parameters describing aspects of the network architecture that can be adjusted to optimize performance independent of final training of the network on training data. Examples include the number of nodes and layers, learning rate and penalty values.
*Overfitting*: Use of an overly complex model by including an excessive number of fitable parameters, allowing very good performance on training data at the cost of loss of generalizability to new test data.
*Saliency mapping*: ML tool similar to backpropagation that allow one to highlight those parts of the input data whose pixels have the greatest influence on the class (outcome) scores.
*Sparse data*: Used here in the specific sense that training data are available in numbers that limit the power of typical training methods, often requiring adjunctive methods such as transfer learning.
*Supervised learning*: Training of a model in which labeled input data (reflecting the ground truth) is compared to the output data predicted by the model in order to optimize its performance.
*Neural net training*: Adjustment of parameters (e.g. weights) of a neural network to optimize its performance on labeled input data for tasks such as class labeling, feature identification, etc.
*Transfer learning*: ML technique whereby a neural net is trained on a (typically larger) set not directly related to the ultimate target set but in some way containing features relevant to the target, followed by retraining (possibly just of the later layers of the network) on target data. It does not “waste” valuable data for primary layer training.
*Weighting*: Multiplicative factors applied to values of multiple inputs to a node to give them different strengths of contribution to the overall node activation. May have a fixed bias added to the sum.
