# Supplementary material for: A brief review of some artificial intelligence methods in nephrology
Source: Pediatr Nephrol. 2025 Oct 27;41(6):1589–98. doi: 10.1007/s00467-025-06995-9 (PMC13139217; doi:10.1007/s00467-025-06995-9)
Supplement: Supplementary file 1 — Graphical abstract (PPTX 140 KB) [file 467_2025_6995_MOESM1_ESM.pptx]

## Slide 1
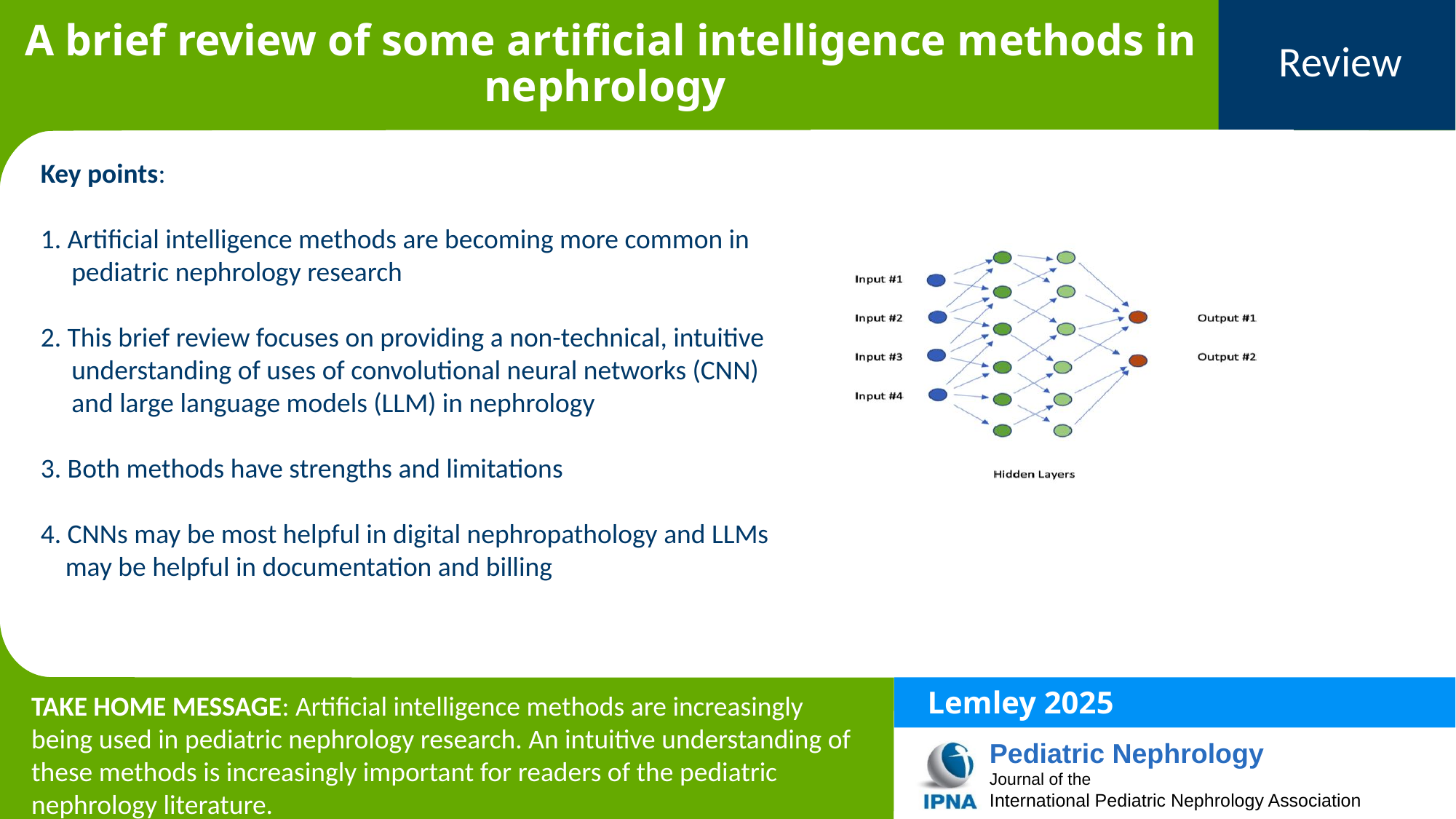

A brief review of some artificial intelligence methods in nephrology
Key points:
1. Artificial intelligence methods are becoming more common in
 pediatric nephrology research
2. This brief review focuses on providing a non-technical, intuitive
 understanding of uses of convolutional neural networks (CNN)
 and large language models (LLM) in nephrology
3. Both methods have strengths and limitations
4. CNNs may be most helpful in digital nephropathology and LLMs
 may be helpful in documentation and billing
Consider including a representative figure or table from your Review article, if relevant, and if you have the requisite permissions.
Lemley 2025
TAKE HOME MESSAGE: Artificial intelligence methods are increasingly being used in pediatric nephrology research. An intuitive understanding of these methods is increasingly important for readers of the pediatric nephrology literature.
-----
